# What are the implications of the spontaneous spleno-renal shunts in liver cirrhosis?

**DOI:** 10.1186/1471-230X-9-89

**Published:** 2009-11-24

**Authors:** Giovanni Tarantino, Vincenzo Citro, Paolo Conca, Antonio Riccio, Marianna Tarantino, Domenico Capone, Michele Cirillo, Roberto Lobello, Vittorio Iaccarino

**Affiliations:** 1 Department of Clinical and Experimental Medicine, Federico II University Medical School of Naples, Naples, Italy; 2 Hepatology Unit of General Medicine, "Mauro Scarlato" Hospital, Scafati, ASL SA/1, Scafati, Italy; 3 Department of Biomorphological and Functional Sciences, Federico II University Medical School of Naples, Naples, Italy; 4 Department of Neurosciences, Unit of Clinical Pharmacology, Federico II University Medical School of Naples, Naples, Italy; 5 Department of Oncology and Endocrinology, Gastrointestinal Surgery Unit, Federico II University Medical School of Naples, Naples, Italy

## Abstract

**Background:**

Although significant advances are expected to be made in the assessment of the portal hypertension-related complications, the prognostic role of spleno-renal shunts has not been fully explored so far. Clarifying this aspect could help tackle the life-treating events occurring in patients suffering from liver cirrhosis. The aim of the study was to analyze the relationships between the spleno-renal shunts presence at doppler ultrasound and the liver cirrhosis complications.

**Methods:**

Design: eighty one patients out of 129 formed the study population (35 females). Chronic liver damage in these patients was caused by HCV (66), HBV (2), alcohol abuse (2) or unknown etiology, likely non-alcoholic steatohepatitis (11). Setting: two Liver Units of university/primary hospitals in Southern Italy. Main outcome measures: grading of esofageal varices; detection of ascites: assessment of hepatic encephalopathy; evaluation of liver cirrhosis severity; tracking hepatocellular carcinoma; doppler features of spleno-renal shunts and splenic flow velocity; spleen longitudinal diameter at sonography.

**Results:**

The prevalence of spleno-renal shunts was 18.5%, without no difference concerning the etiology (HCV versus non-HCV, p = 0.870); the prevalence of hepatocellular carcinoma in patients with spleno-renal shunts was superior to that of patients without them (Pearson Chi-square, p = 0.006, power of sample size 74%), also after adjustment for liver decompensation (p = 0.024). The median score of hepatic encephalopathy in patients with and without spleno-renal shunts was similar, i.e., 0 (range, 0-2) versus 0 (0 - 3), p = 0.67. The median splenic vein flow velocity in patients with spleno-renal shunts was significantly inferior to that of patients without them, i.e., 13 cm/sec (95% confidence intervals, 6-18) versus 21 cm/sec (17-24), p < 0.0001. By far the largest percentage of large esophageal varices was in patients without spleno-renal shunts (p = 0.005). In contrast, the frequency of ascites and hepatic encephalopathy severity was overlapping in the two groups. BMI values but not Child-Pugh's classification predicted spleno-renal shunts (Ors = 1.84, 95% confidence intervals = 1.28-2.64, p = 0.001 and 1.145, 95% confidence intervals = 0.77-1.51, p = 0.66).

**Conclusion:**

Taking into consideration the relatively small sample size, patients with spleno-renal shunts are burdened by an increased incidence of hepatocellular carcinoma. BMI predicted the spleno-renal shunts presence.

## Background

The understanding of mechanisms regulating Portal Hypertension (PH) has been incomplete so far, although researchers have struggled for years. PH is primarily caused by an increase in resistance to portal outflow and secondly by a growth in splanchnic blood flow [1]. In a later phase, these changes lead to a hyperkinetic circulation that raises cardiac output and reduces systemic vascular resistance as well as perfusion pressure. Regional alterations in vasoreactivity (vasodilation and vasoconstriction), sinusoidal remodelling and capillarization, angiogenesis, venous thrombosis, and obviously vascular distortion, all play a certain role in the pathophysiology of PH by contributing to circulatory impairment and expansion of the collateral circulation. Among vasoactive substances activated in PH, nitric oxide is considered as the most important vasodilator. Endothelin-1 and cyclooxygenase-derived prostaglandins are the foremost vasoconstrictor factors, apart from the sympathetic overactivity. Furthermore, a major role has been attributed to activated hypercontractile hepatic stellate cells, which cause vascular remodelling as an adaptive response to the changed balance in vasoactive substances. It has recently demonstrated that endothelial dysfunction is determinant in PH development. Finally, an increase in the splanchnic vascular bed size mediated by a Vascular Endothelial Growth Factor (VEGF)-dependent angiogenic process has been claimed to significantly contribute to increased overall blood flow in splanchnic tissues of PH animals. In addition, this VEGF-dependent angiogenesis also plays a crucial role in the formation of portal-systemic collateral vessels, which include Spleno-Renal shunts (SRS [2]). Notably, novel research has highlighted that PH is an independent predictor of HepatoCellular Carcinoma (HCC) development [3]. Beyond the formation of portosystemic shunts, of which Esophageal Varices (EV) have the greatest clinical impact and the most severe complications, PH leads to Hepatic Encephalopathy (HE [4]) and ascites [5]. Other manifestations of PH include portal hypertensive gastropathy [6] and Large Spontaneous Shunts (LSS [7]). LSS refer to the presence of patent paraumbilical vein, SRS, ano-rectosigmoid varices with or without portal hypertensive colopathy. The prevalence of umbilical vein patency ranges from 6% to 30% in patients with PH [8]. SRS are present in cirrhotics from nearly 14% up to 21% [9,10]. Rectosigmoid varices are present in nearly one third of cirrhotic patients, 4% of whom have a lower gastrointestinal hemorrhage [11]. LSS, the presence of which was assessed by portal venous phase multidetector-row spiral computed tomography, have been shown to be responsible for recurrent or persistent HE [12]. In patients with liver cirrhosis, with and without bleeding varices, many imaging techniques are available to detect collaterals, beyond the previously quoted one. In fact, Magnetic Resonance Imaging (MRI [13]), single photon emission computed tomography [14,15], selective intra-arterial digital subtraction angiography [16], scintiphotosplenoportography [17], but also the percutaneous transhepatic portography [18], are quite conclusive in diagnosing SRS. Reports published in the literature to date have shown that all patients with hepatic cirrhosis should be studied by using Doppler UltraSound (DUS) techniques [19], being the remainder methods invasive or extremely expensive and then unsuitable in close follow-ups. DUS can provide a great deal of information about the morphology and hemodynamics of PH and consequently spontaneous shunts can be identified [20]. They may mimic surgically created shunts in their large volume-flow capacity. In contrast, other Authors believe that hepato-splenic hemodynamics, as studied at DUS, are only weakly correlated to portal pressure, suggesting measuring them with MRI [21]. Anyway, among blood flow parameters, Splenic Vein Flow Velocity (SVFV) is considered to be specifically related to the stages of liver damage [22] and to EV bleeding [23]. Although significant advances are expected to be made in the diagnosis and management of the PH-related complications, in the face of an increasing burden of chronic liver disease, the prognostic role of LSS has not been presented in a convincing manner so far. Clarifying this aspect could help correctly approach the ongoing, life-treating events occurring in patients suffering from liver cirrhosis. Therefore, the objective of this study was to evaluate the relation of SRS, detected by an easily reproducible and reliable tool, i.e., DUS, to the severity of ascites, EV, HE and the presence of HCC. Furthermore, we intended to seek any association of these imaging parameters with the degree of liver failure.

## Methods

This research was performed screening 129 consecutive patients with established liver cirrhosis admitted to two Liver Units of university/primary hospitals in Southern Italy from October 2006 to December 2008. The study was carried out according to the principles of the Declaration of Helsinki and an informed written consent was obtained from each patient.

### Exclusion criteria

Out of the initial patients, 23 were excluded because their instrumental examinations (DUS) had been previously performed in different centres. Fourteen patients, who had undergone endoscopic EV ligation therapy, and eight, who had received beta-blockers, were also disallowed from the study because prior treatment might have caused a change in DUS features. Three patients were left out for the detection of thrombosis of the portal vein.

### Inclusion criteria

The remaining 81 patients formed the study population (35 females) whose age was 68.2, 8.18, 66.4-70 years (mean, +/-SD, 95% CI). Chronic liver damage in these patients was caused by hepatitis C (n = 66), hepatitis B (n = 2), alcohol abuse (n = 2) or unknown etiology, likely Non-Alcoholic SteatoHepatitis (NASH) (n = 11). Forty seven patients had compensated cirrhosis of the liver. For 71 patients, the diagnosis of cirrhosis was established by contextual clinical (spider nevi, hepato-splenomegaly), laboratory (low serum total cholesterol, prothrombin activity and pseudocholinesterase levels, reduced white blood cell and platelets count, globulin/albumin ratio >1,[24]) as well as antecedent imaging data. Being these parameters inadequate to confirm the diagnosis in 10 patients, successively assigned to the Child's class A, a liver biopsy was performed. The non-invasive assessment of liver cirrhosis was blindly performed *de novo *to all patients by experts on the basis of UltraSound (US) and DUS examinations (coarse echo-texture, nodularity presence, increased caudate/right lobe ratio, hypertrophy of the left lobe, characterized by a rounded inferior marginal edge, and portal vein enlargement with decreased flow velocity, absence of a normal doppler waveform, hepatofugal flow). Alcohol abuse was diagnosed according to the DSM-IV criteria, by means of screening tests such as MAST (Michigan Alcohol Screening Test) and CAGE (Cut down, Annoyed, Guilty, and Eye opener), as well as random tests for blood alcohol concentration and the use of a surrogate marker, e.g., Mean Corpuscular Volume.

Prestudy agreement, shared by all the investigators, gave place to common diagnostic procedures in order to meet the target of concordance. Patients should have fulfilled the following criterion at entry, i.e., US/DUS, endoscopic and laboratory examinations performed within four weeks of each other.

### Endoscopy

EV were graded according to a previous classification, i.e., F1 small and straight; F2 moderately sized, tortuous, and occupying less than one third of the lumen; F3 large, coiled, and occupying one third or more of the lumen [25]. Large EV (LEV) were considered F2 and F3.

### Ascites presence at ultrasound

The superior end of the right paracolic gutter and the pelvis were carefully assessed at US. Small quantities were sought for around the liver or spleen surface and in the Morrison's pouch [26]. The presence of peritoneo-pleural communications was determined. BMI of patients with ascites was adjusted taking into consideration the weight before this complication.

### Hepatic encephalopathy grade

HE was graded based on the level of consciousness, intellectual functions, behaviour and neuromuscular functions according to West Haven (W-H) criteria [27]. W-H grade 0 or minimal encephalopathy was ascertained as previously described [28].

### Assessment of liver cirrhosis severity

In all patients the severity of illness was assessed using a modified Child-Pugh's classification [29], Table 1). The decompesation criterion was set at score 8 of Child-Pugh's classification [30], although some patients presented light ascites or minimal encephalopathy under this cut-off.

**Table 1:** Child-Pugh classification slightly modified.

Clinic and laboratory data	Points for increasing abnormality^1^		
	1	2	*3*
HE (grade^2^)	None	SHE (0), 1-2	3-4
Ascites	None at US	Mild or controlled by diuretics	Present despite diuretics
PT (% of activity)^3^	> 70	70-40	< 40
Albumin (g/dL)	> 3.5	2.8-3.5	< 2.8
Bilirubin (mg/dL)	< 2	2-3	> 3

^1^Scoring system: 5-6 points, grade A; 7-9 points, grade B; 10-15 points, grade C. ^2^HE: hepatic encephalopathy; Grade 0: sub-clinical hepatic encephalopathy, SHE, unravelled by Retain tests A or B; Grade 1: anxiety, irritability, depression, impaired concentration, sleep disturbances; Grade 2: disorientation, poor short-term memory, disinhibited behavior, drowsiness; Grade 3: somnolence, bizarre behavior, confusion, amnesia, paranoia; Grade 4: Coma. ^3^PT, Prothrombin Time, also expressed in seconds prolonged < 4; 4-6; > 6, or as INR < 1.7; 1.7-2.3; > 2.3. US: UltraSound.

### Hepatocellular carcinoma detection

Patients were screened for HCC by performing abdominal US and testing alpha-fetoprotein at admission, in course of regular 6-monthly or annually (by computed tomography) surveillance. When a surveillance feature or test was abnormal (>10.9 ng/mL), a triple-phase imaging (MRI) was recommended for evaluation at recall.

### Ultrasound and Doppler ultrasound features of spleno-renalshunts

The SRS detection was based on the following features:

Evidence of tortuous, inferiorly directed vessels from the splenic hilum to the left kidney (when dealing with the planes of orientation, i.e., coronal, horizontal, and sagittal, they were primarily seen on the coronal ones);

Splenofugal blood flow [31];

Dilatation of the left renal vein with blood flow phasic and at high velocity (≥20 cm/sec);

Marked increase of the spleen volume (see below);

Dilatation of the splenic vein diameter (≥5 mm) with blood flow phasic and at high velocity (SVFV ≥15 cm/sec);

#### Hepatofugal blood flow in the portal vein

Spleen Longitudinal Diameter (SLD) was performed by postero-lateral scanning. The Maximum Length (ML, the optically greatest overall longitudinal dimension obtained from one of the two poles) and the Cranio-Caudal Length (CCL, the optically maximal transversal dimension intercepting one of the two poles) were measured; the resulting values were then averaged, since the two measurements do not always coincide. SLD, (ML + CCL/2), ≥150 mm made the spleen be considered definitely enlarged, being this parameter best correlated to its size.

### Statistics

BMI, SVFV and Child-Pugh's classification values, not normally distributed (Shapiro-Wilk test, p = 0.001, 0.001 and 0.01), were expressed as median (range). HE, EV and ascites grades were considered ordinals and managed in the same way. The difference in medians was assessed by the Kolmogorov-Smirnov (K-S) Two-Sample test. Age data, derived from a normally distributed population (Shapiro-Wilk test, p = 0.44), were articulated as mean plus SD and 95% confidence intervals (CI); the difference of means was evaluated by Two-Sample t test. The Two-Way Tables cross-tabulated one or more than one categorical row variables with one categorical column variable and the significance was set by the Pearson Chi-square. When cross-tabulation was stratified for another dichotomous variable the Mantel-Haenszel Chi-square was carried out. The correlation between Child-Pugh's scores and SVFV values was determined by the Spearman's rho. To make prediction the logistic regression (Enter method) was adopted, selecting as independent variable BMI values as well as the Child-Pugh classification and as dependent variable SRS presence/absence; HE and ascites severity were not tested in the context of the previous independent variables to avoid multi-collinearity (situation in which the predictors are correlated to each other to some degree). To assess the independent effect of a quantitative variable, i.e., SFV, on the prediction of the ascites, HE and EV grades the linear regression analysis (least squares) was used, evaluating the standardized coefficient beta (β). The Factor Analysis was applied to detect the structure in the relationships among variables selecting a subset of variables, which have the highest correlations with the principal component factors. The Cattel Scree plot, with relative eigenvalues, was performed to screen the real factors. Extraction of the main components amounted to a variance maximizing (varimax) rotation of the original variable space. The critical value was calculated by the formula: doubling the Pearson's correlation coefficient for 1% level of significance (5.152)/square root of patients minus 2, i.e., 0.579. The concordance correlation coefficient (ρ_c_), which measures precision and accuracy, was adopted to evaluate the degree of pair observations at US. The power of our sample size was calculated by the non-equality of proportion with a type I error, alpha, of 0.05. Statistical analysis was performed operating on Systat 12 (Richmond, CA, USA) and MedCalc Version 11^® ^(Frank Schoonjans) software packages.

## Results

In order to allow readers to gauge how well the study findings apply to their patients (external validity) we stress that eighty one patients, divided into two groups of, i.e., those with and without SRS, were well balanced in respect to severity of disease (p = 0.51, Table 2), gender (p = 0.76) and age (p = 0.98). The median score of W-H criteria in patients with and without SRS was similar, i.e., 0 (0-2) versus 0 (0-3), p = 0.67. The median value of SVFV in patients without SRS was significantly inferior to that of patients with SRS, i.e., 13 cm/sec [6-18] versus 21 cm/sec [17 - 24], p < 0.0001.

**Table 2:** Spleno-renal shunts distribution through liver cirrhosis severity.

CHILD Score	SRS Absent	SRS Present	Total
5	9	1	10

6	13	2	15

7	16	6	22

8	13	1	14

9	7	3	10

10	4	0	4

11	3	1	4

12	1	1	2

Total	66	15	81

SRS, Spleno-renal Shunts; Pearson Chi-square, p = 0.51.

### Prevalence

The prevalence of SRS in our patients, screened at DUS, was exactly 18.5% without any difference concerning the etiology of liver cirrhosis (HCV versus non-HCV, Pearson Chi-square p = 0.870). Ascites, LEV and HE were present in 28.3%, 38.3% and 24.7% of our population, respectively. By far the largest percentage of LEV was in patients without SRS (Pearson Chi-square, p = 0.005, Table 3). In contrast, the frequency of HE severity and ascites was overlapping (Tables 4 and 5). The prevalence of HCC in patients with SRS was superior to that of patients without it (Pearson Chi-square, p = 0.006, Table 6), also after adjustment for liver decompensation (Mantel-Haenszel Chi-square, p = 0.024). HCC, equally detected on the basis of the gender (Pearson Chi-square, p = 0.436), counted for a part (8.6%) of our cohort according to the expected incidence.

**Table 3:** Spleno-renal shunts distribution through large esofageal varices presence.

LEV	Absent	Present	Total
SRS Absent	36	30	66
SRS Present	14	1	15
Total	50	31	81

LEV, Large Esofageal Varices; SRS, Spleno-Renal Shunts; Pearson Chi-square, p = 0.005.

**Table 4:** Spleno-renal shunts distribution through hepatic encephalopathy severity.

HE Grade	Absent or Minimal	1	2	3	Total
SRS Absent	50	9	6	1	66
SRS Present	10	4	1	0	15
Total	60	13	7	1	81

SRS, Spleno-Renal Shunts; HE, Hepatic Encephalopathy; Pearson Chi-square, p = 0. 63.

**Table 5:** Spleno-renal shunts distribution through ascites severity.

Ascites	Absent	Grade 1	Grade 2	Total
SRS Absent	46	13	7	66
SRS Present	12	2	1	15
Total	58	15	8	81

SRS, Spleno-Renal Shunts; Pearson Chi-square, p = 0.73.

**Table 6:** Spleno-renal shunts distribution through hepatocellular carcinoma presence

HCC	Absent	Present	Total
SRS Absent	63	3	66
SRS Present	11	4	15
Total	74	7	81

SRS, Spleno-Renal Shunts; HCC, HepatoCellular Carcinoma; Pearson Chi-square, p = 0.006.

### Association

The correlation of the studied DUS parameter, i.e., SVFV, to the degree of liver failure, weighed by Child-Pugh's scores was inversely significant (rho = -0.430, p = 0.001).

**Figure 1:**
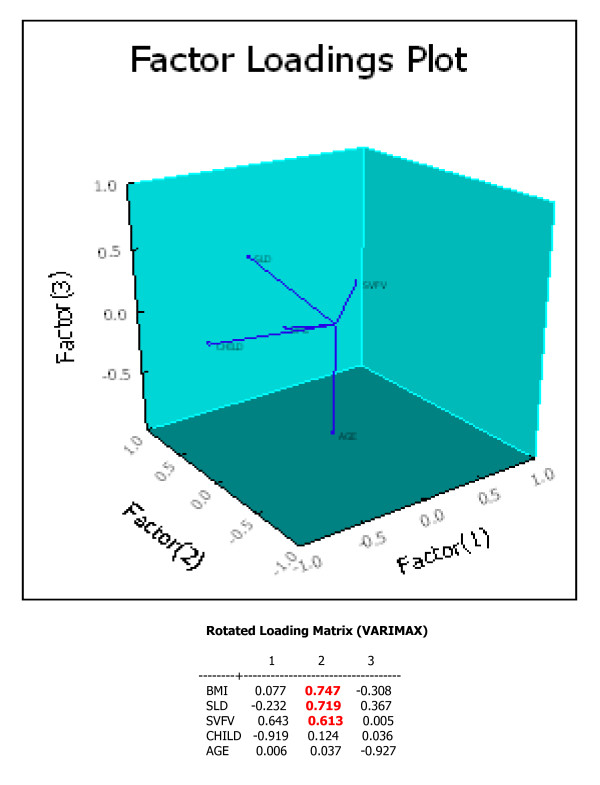
**Factor Loadings Plot**. Body Mass Index, BMI; Spleen Longitudinal Diameter, SLD; Splenic Vein Flow velocity, SVFV. The highest loading is on Factor 2, where three variables are strictly correlated.

Interestingly, a hidden correlation was found among the SLD, the SVFV and the BMI, independently from age of patients and perhaps of the illness, Figure 1.

### Prediction

The estimates of Child-Pugh's classification and BMI values in predicting the SRS presence gave the following results: OR = 1.145, 95% CI = 0.77 - 1.51, p = 0.66 and OR = 1.84, 95% CI = 1.28-2.64, p = 0.001, respectively. Lower SVFV values predicted more advanced ascites, HE and EV grades (β = -0.29, p = 0.009; β = -0.37, p = 0.001 and β = -0.41, p = 0.0001, respectively).

### Accuracy

The agreement of US paired observations ranked high, i.e., ρ_c _= 0.90.

### Power analysis

When analysing the impact of the sample size regarding the HCC presence through the SRS distribution, the power was 74% with a type I error of 0.05.

### Discussion

The essential findings we provide in this study are briefly i) greater occurrence of HCC in patients with SRS than in those without them; ii) clear prediction of BMI towards the SRS presence; iii) detection of fewer LEV in patients presenting SRS; iiii) same prevalence of ascites and HE presence in patients with and without SRS. Although knowledge regarding mechanisms involved in the pathogenesis of PH has taken unprecedented levels, nevertheless many prognostic aspects still remain to be elucidated. If one considers that knowing them could help better manage cirrhotic patients, it would then be justifiable to speculate whether physicians could predict the history of liver cirrhosis on the basis of the spontaneous SRS presence by some means. This study aimed at partially answering this question. The body of present knowledge [9] is in favour of the fact that the presence of portosystemic collaterals identifies cirrhotics with a less favourable clinical course. Our data only partially agree with this conclusion. In fact, we provide evidence for a low risk of LEV and a quite similar prevalence of HE and ascites in patients with SRS. However, the procrastination of haemorrhagic risk from LEV in SRS patients would be only temporary, because the basal critical PH identifies a more severe grade of liver cirrhosis, i.e., very high and long-lasting sinusoidal resistance.

Some of our results are expected if the following is examined. A recent study comparing Endoscopic Sclerotherapy (ES) with distal surgical portosystemic shunts in the prevention of recurrent variceal bleeding, has showed that both these interventions achieved a positive effect on variceal rebleeding [32]. A switch to decompressive shunt procedures is mandatory in case of ES or Transjugular Intrahepatic Portosystemic Shunt (TIPS) insertion fails to control recurrent variceal hemorrhage [33]. Selective shunts are placed surgically to manage post-transplant portal vein stenosis/thrombosis. In contrast, preexisting spontaneous portosystemic shunts increase the risk of post-transplantation portal vein thrombosis [34] and hamper graft survival [35]. Furthermore, SRS main disadvantage is decreased perfusion of the liver with eventually reduction in liver volume and related function [36]. Nevertheless, surgeons are convinced that the creation of peripheral portosystemic shunt still has a role in the treatment of some patients with PH on the basis that long-term blood flow in the portal vein was not severely reduced after this intervention [37]. Finally, a meta-analysis of individual patient data has provided further evidence that TIPS significantly impacts only on those patients with the extreme grade of ascites [38], although it worsens encephalopathy [39]. The novelty of our study relies on the evidence of an interesting percentage of patients with SRS in which HCC has been detected, mainly taking into consideration that HCC prevalence in our population was lower than that found among Japanese patients (confined to HCV infection), obtained summarizing four representative studies [40], which was 16% (212/1339). But, how can we explain this major occurrence of HCC in them? We emphasize that HCC, the third leading cause of cancer mortality worldwide, is a highly vascular tumor that expresses VEGF [41]. An up-to-date study provides further evidence that obesity increases HCC risk and that this factor may explain a relevant proportion of cases among subjects in absence of HBV/HCV infection, probably NASH-mediated [42]. Interestingly, patients suffering from NASH, a further expression of the metabolic syndrome, express high serum concentrations of VEGF [43]. Finally, portal VEGF was significantly higher than systemic VEGF, and expressions of VEGF and hepatocyte growth factor in the liver, spleen and intestine were also up-regulated during liver regeneration [44].

In term of clinical advantage, which take-home lesson could be drawn from our data? Physicians should try to strike a balance between pprogressive nutrition deficiencies as well as muscle wasting, universal problems in patients suffering from liver cirrhosis, and a moderately hypocaloric diet to avoid this eventual risk factor.

As it is repeatedly emphasized, PH remains a partially-clarified phenomenon and so does the collaterals presence. Now, the VEGF-dependent angiogenesis is considered being crucial in determining SRS [2]. A fascinating hypothesis suggests that portosystemic shunts may mimic an Arterio-Venous Fistula (A-VF [45]), in which the high-pressure portal blood relays with the lower pressure systemic venous circulation. Although these collaterals decompress the portal circulation, the increased cardiac output enhances portal blood flow and tends to counteract the portal hypotensive effect of the portosystemic shunt. As portal blood flow grows, collateral blood flow multiplies and is nearly totally shunted in the systemic circulation. Ultimately, high-output cardiac failure occurs leading to cirrhotic cardiomyopathy. Actually, researchers demonstrated an over-expression of VEGF in a rabbit A-VF model [46].

Coming back to the HE detection in both patients with and without SRS, what does support this finding? In contrast with the current opinion, a recent study in patients with cirrhosis has failed to find a relationship between EEG alterations and the presence per se of a patent paraumbilical vein, very common collateral with the same significance of SRS [7]. Comparing angiographically patients evidencing LSS and HE with cases characterized by PH but no HE, Takashi et al. concluded that small SRS were not associated with HE [47]. On the other hand, some patients with spontaneous shunts, who had undergone shunt reduction by a radiological approach [48], obtained an amelioration of HE. In conclusion, SRS could only provide an explanation for the refractoriness of HE. Continuing on the subject, the lack of association between liver cirrhosis severity and SRS, found in our series, is confirmed by the subsequent observations. Following-up consecutive patients with bleeding varices and LSS, after devascularization with or without esophageal transaction, the postoperative survival rate of patients with the LSS was significantly lower than that of patients without the LSS and preoperative variables concerning hepatic reserve failed to show significant predictability [16]. Other data challenge this finding [9]. Discussing the possible limitations of the present study we have to pinpoint that the sample size of patients with HCC is small, but it seems to be of sufficient power and adequate to the low (5%) incidence of this complication in liver cirrhosis [49]. Moreover, it is correct to evidence that other cutting-edge technology is more specific for the detection of portosystemic collaterals, e.g., MRI [13]. But, any crucial future research directions should consider the utility of DUS particularly when repeated measures are requested beyond liver transplantation settings [34,35]. Last but not least consideration, are we sure that splenomegaly is the consequence of PH and not one of its causes, mainly taking into consideration the spleno-renal reflex-mediated reduction in vascular conductance [50] that exacerbates sodium and water retention in the kidneys?

## Conclusion

SRS in liver cirrhosis is a feature more neglected in clinical practice than among imaging operators. Forecasting whether the search for SRS would be carried out years down the line is a dodgy business, because this attitude is not popular with physicians even though it could influence the prognostic choices. What will the positive and negative (in respect to the moderately increased costs) effects be, it is necessary to ascertain. Anyway, patients with evidence of SRS are burdened by an enhanced incidence of HCC, mainly if they are overweight or obese. The awareness of this crucial complication should increase in order to bring a survival advantage to those patients.

## Abbreviation list

PH: Portal Hypertension; EV: Esophageal Varices; HE: HepaticEncephalopathy; LSS: Large Spontaneous Shunts; SRS: Spleno-RenalShunts; US: UltraSound; MRI: Magnetic Resonance Imaging; HCC:HepatoCellular Carcinoma; VEGF: Vascular Endothelial Growth Factor; DUS: Doppler UltraSound; TIPS: Transjugular Intrahepatic Portosystemic Shunt; SVFV: Splenic Vein Flow Velocity; LEV: Large EV; ES: Endoscopic Sclerotherapy; W-H: West Haven; ML: Maximum Length; CCL: Cranio-Caudal Length; SLD: Spleen Longitudinal Diameter; β: standardized coefficient beta; ρ_c_: correlationcoefficient.

## Competing interests

The authors declare that they have no competing interests.

## Authors' contributions

TG conceived of the study and drafted the manuscript. CV and CP carried out the clinical investigation. IV participated in the design of the study, and RA and CD helped to draft the manuscript. TM and TG performed ultrasound. TG performed the statistical analysis. CM and LR made endoscopy. All authors read and approved the final manuscript.

## Pre-publication history

The pre-publication history for this paper can be accessed here:

http://www.biomedcentral.com/1471-230X/9/89/prepub
